# Oncological and Survival Endpoints in Cancer Cachexia Clinical Trials: Systematic Review 6 of the Cachexia Endpoints Series

**DOI:** 10.1002/jcsm.13756

**Published:** 2025-03-10

**Authors:** Olav Dajani, Iain Philips, Ester Kristine Størkson, Trude R. Balstad, Leo R. Brown, Asta Bye, Ross Dolan, Christine Greil, Marianne Hjermstad, Gunnhild Jakobsen, Stein Kaasa, James McDonald, Inger Ottestad, Judith Sayers, Melanie Simpson, Mariana S. Sousa, Ola Magne Vagnildhaug, Michael S. Yule, Barry J. A. Laird, Richard J. E. Skipworth, Tora S. Solheim, Mark Stares, Jann Arends, the Cancer Cachexia Endpoints Working Group

**Affiliations:** ^1^ Regional Advisory Unit for Palliative Care, Dept. of Oncology, Oslo University Hospital/European Palliative Care Research Centre (PRC), Dept. of Oncology, Oslo University Hospital, and Institute of Clinical Medicine University of Oslo Oslo Norway; ^2^ Edinburgh Cancer Research Centre, Institute of Genetics and Cancer University of Edinburgh Edinburgh UK; ^3^ Department of Clinical Medicine, Clinical Nutrition Research Group UiT The Arctic University of Norway Tromsø Norway; ^4^ Department of Clinical and Molecular Medicine, Faculty of Medicine and Health Sciences NTNU ‐ Norwegian University of Science and Technology Trondheim Norway; ^5^ Royal Infirmary of Edinburgh Clinical Surgery University of Edinburgh Edinburgh UK; ^6^ Department of Nursing and Health Promotion, Faculty of Health Sciences Oslo Metropolitan University Oslo Norway; ^7^ Academic Unit of Surgery University of Glasgow, Glasgow Royal Infirmary Glasgow UK; ^8^ Department of Medicine I, Medical Center ‐ University of Freiburg, Faculty of Medicine University of Freiburg Freiburg Germany; ^9^ Department of Public Health and Nursing Norwegian University of Science and Technology Trondheim Norway; ^10^ Department of Nutrition, Institute of Basic Medical Sciences, Faculty of Medicine University of Oslo Oslo Norway; ^11^ The Clinical Nutrition Outpatient Clinic, Section of Clinical Nutrition, Department of Clinical Service, Division of Cancer Medicine Oslo University Hospital Oslo Norway; ^12^ Palliative Care St Columba's Hospice Care Edinburgh UK; ^13^ Improving Palliative, Aged and Chronic Care Through Clinical Research and Translation (IMPACCT) University of Technology Sydney Sydney Australia; ^14^ Cancer Clinic St Olav's Hospital ‐ Trondheim University Hospital Trondheim Norway

**Keywords:** adverse events, cachexia, cancer, clinical trials, survival

## Abstract

**Background:**

In patients receiving anti‐cancer treatment, cachexia results in poorer oncological outcomes. However, there is limited understanding and no systematic review of oncological endpoints in cancer cachexia (CC) trials. This review examines oncological endpoints in CC clinical trials.

**Methods:**

An electronic literature search of MEDLINE, Embase and Cochrane databases (1990–2023) was performed. Eligibility criteria comprised participants ≥ 18 years old; controlled design; ≥ 40 participants; and a cachexia intervention for > 14 days. Trials reporting at least one oncological endpoint were selected for analysis. Data extraction was performed using Covidence and followed PRISMA guidelines and the review was registered (PROSPERO CRD42022276710).

**Results:**

Fifty‐seven trials were eligible, totalling 9743 patients (median: 107, IQR: 173). Twenty‐six (46%) trials focussed on a single tumour site: eight in lung, six in pancreatic, six in head and neck and six in GI cancers. Forty‐two (74%) studies included patients with Stage III/IV disease, and 41 (70%) included patients receiving palliative anti‐cancer treatment. Ten studies (18%) involved patients on curative treatment. Twenty‐eight (49%) studies used pharmacological interventions, 29 (50%) used oral nutrition, and two (4%) used enteral or parenteral nutrition. Reported oncological endpoints included overall survival (OS, *n* = 46 trials), progression‐free survival (PFS, *n* = 7), duration of response (DR, *n* = 1), response rate (RR, *n* = 9), completion of treatment (TC, *n* = 11) and toxicity/adverse events (AE, *n* = 42). Median OS differed widely from 60 to 3468 days. Of the 46 studies, only three reported a significant positive effect on survival. Seven trials showed a difference in AE, four in TC, one in PFS and one in RR. Reported significances were unreliable due to missing adjustments for extensive multiple testing. Only three of the six trials using OS as the primary endpoint reported pre‐trial sample size calculations, but only one recruited the planned number of patients.

**Conclusion:**

In CC trials, oncological endpoints were mostly secondary and only few significant findings have been reported. Due to heterogeneity in oncological settings, nutritional and metabolic status and interventions, firm conclusions about CC treatment are not possible. OS and AE are relevant endpoints, but future trials targeting clinically meaningful hazard ratios will required more homogeneous patient cohorts, adequate pre‐trial power analyses and adherence to statistical testing standards.

## 1 Introduction

Cancer cachexia is a complex syndrome characterized by an ongoing loss of skeletal muscle mass that cannot be fully reversed by conventional nutritional support and leads to progressive functional impairment [1]. Cachexia develops frequently in patients with advanced cancer and is associated not only with distressing symptoms and reduced quality of life (QoL) but also with poor clinical outcomes. Cachexia clinical trials have sought to improve food intake, body composition (muscle) and physical function to alleviate symptoms and improve QoL. However, it is reasonable to question whether anti‐cachexia treatments, either by improving tolerance to anti‐cancer treatment or by potentially modifying the tumour response, also might have an effect on relevant oncological outcomes such as response to cancer therapy or overall survival (OS).

To answer this question is challenging as progress in cachexia research has been impeded by the absence of universally accepted endpoints for interventional cancer cachexia trials. Endpoints targeted in clinical trials are diverse and belong to separate domains. Some of these domains have been reviewed recently, including QoL [2], appetite and dietary intake [3], physical function [4], body weight and composition [5] and biomarkers [6]. Below, we complete this appraisal of cachexia endpoints by undertaking an analysis of oncological endpoints reported in randomized cancer cachexia trials.

Oncological endpoints targeted in trials studying patients with solid tumours or haematological malignancies are heterogeneous, including overall and milestone survival, endpoints focusing on the duration of a measurable benefit (progression‐free survival [PFS], time to progression, event‐free survival, disease‐free survival, time to treatment failure, time to next treatment, duration of clinical benefit and duration of response) and endpoints focusing on the rate of a measurable benefit (overall, complete response and pathological complete response rate (RR), disease control rate and clinical benefit rate) [7]. We acknowledge that survival is not only an oncological outcome but also a key cachexia endpoint. It is also important to assess adverse events (AEs) as endpoints [8], given they are essential to monitoring safety in clinical trials as suggested by the European Medicines Agency (EMA). In addition, in the context of anti‐cancer treatments, supportive care measures have been judged by their effect on anti‐cancer treatment dosing. It is well known that interruptions of anti‐cancer treatment, delayed treatment or reduction in treatment intensity is associated with poorer outcome, especially in patients with potentially curable malignancies [9, 10, 11].

The main objective of this systematic review was to describe the frequency and variety of oncological endpoints reported in cancer cachexia trials. This review includes descriptions of trial characteristics, interventions, choices and reporting of oncological endpoints and data quality of reported oncological endpoints.

## 2 Methods

This systematic review is one of six reviews examining different endpoints in cachexia (physical function, appetite and dietary intake, QoL, body weight and composition, biomarkers and oncological). These reviews shared a common, overall search strategy, and data extraction was done en masse. It was conducted according to the Preferred Reporting for Systematic Reviews and Meta‐Analyses (PRISMA) statement [12], and it was registered on the International Prospective Register of Systematic Reviews (PROSPERO CRD42022276710) where further detail is available [13].

The search for studies published from 1 January 1990 until 17 October 2023 was conducted by a research librarian (University of Oslo, NO) using the databases MEDLINE (Ovid), EMBASE (Ovid) and Cochrane Register of Controlled Trials. A detailed search strategy is outlined in Appendix 1.

### 2.1 Eligibility Criteria

Studies were considered eligible in a first step if they were controlled trials investigating interventions that aimed to treat or attenuate cachexia in adult patients with cancer. There were no restrictions in type of intervention (pharmacological, nutritional, exercise, multimodal, etc.) nor type of comparator. To reduce bias and focus on outcomes with most clinical impact, studies were excluded if they included fewer than 40 patients and/or the intervention lasted fewer than 14 days. Eligible trials were written in English.

### 2.2 Data Selection and Extraction

All studies identified were transferred to Covidence software [14]. Article selection based on titles and abstract was carried out by three independent reviewers (O.D., B.L. and T.S.S.). Any uncertainties in assessing the eligibility of the studies were discussed until a consensus was reached.

A data extraction table was developed, pilot‐tested and refined before data were extracted from each article by two independent authors from the review group. Studies relevant to this systematic review were then identified from the data by full‐text searches of all eligible trials performed by three independent reviewers (O.D., I.P. and J.A.). Finally eligible for this review were those studies, which assessed at least one oncological endpoint as defined by Delgado and Guddati [7] or any AEs.

### 2.3 Assessing Risk of Bias

The methodological quality of each study was systematically assessed by four independent reviewers (J.M., J.S., O.D. and B.L.) using the modified 10‐point Downs and Black checklist [15] with consensus reached through discussion; a score of 10 indicates a study of the highest quality. Among other criteria, the tool assesses study design, external and internal validity, estimate of variance reporting and whether the outcome is defined and robust.

### 2.4 Data Analysis

Outcomes were reported narratively, describing the variety and frequencies of the endpoints. In studies where the sample size was more than 100, raw data on objective measures and corresponding variability of measures were extracted and presented in keeping with PRISMA guidelines [16].

#### 2.4.1 Determining Endpoints

For each trial, we determined the primary endpoint(s) by extracting outcomes on which sample size calculations were based and/or which were declared in the article explicitly to be ‘primary endpoints’ or some analogue wording (e.g., ‘main outcomes’ and ‘primary aim’). If no primary endpoint could be determined, we defined ‘major outcomes’ as outcomes, which were reported in the abstract of the article.

### 2.5 Comparing Survival Data

To compare the effects on OS between the trials, data were interconverted from survival fractions to median survival times according to the exponential model f(t) = h·e^−ht^, where h = hazard rate, survival fraction S(t) = e^−ht^ and median survival = ln(2)/h. Hazard ratios (HR) are the ratios of two hazard rates. Calculation of sample sizes required to detected differences between two survival proportions followed customary calculations based on the normal approximation to the binomial distribution [17, 18].

## 3 Results

**FIGURE 1:**
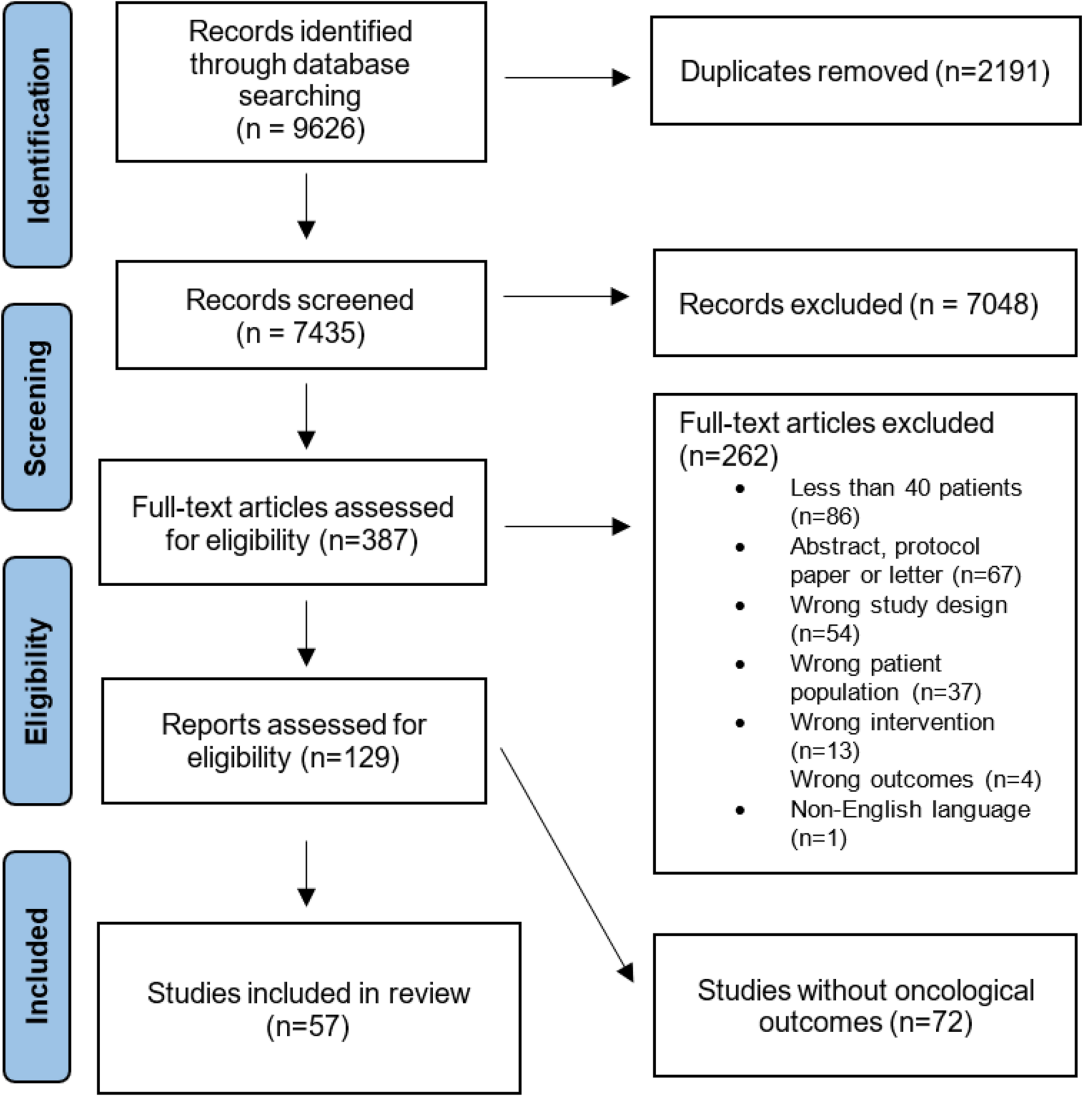
PRISMA diagram.

Figure 1 shows the PRISMA flow chart. After removal of duplicates, 7435 records were reviewed by title (or abstract where the title was insufficient), resulting in 387 records being appraised in full. Of these, 57 studies were finally eligible based on assessing at least one oncological endpoint. Of 16 endpoints defined by Delgado and Guddati [7], the following were found to have been reported in the analysed trials: OS, PFS, duration of response, RR and completion of treatment. AEs were assessed by 42 trials and were the only oncological endpoint in three trials.

Table 1 shows the characteristics of the 57 eligible trials. In total, they included 9743 patients and the sample size varied from *n* = 40 to *n* = 979 (median: 107, IQR: 173). The trials were heterogeneous in terms of intervention, tumour site, tumour stage, oncological treatment setting and nutritional status. Only trials of at least 2 weeks duration were included; the duration of the analysed trials varied from 2 to 104 weeks (median 12, IQR 8–12.75 weeks).

**TABLE 1:** Characteristics of eligible trials.

Article					Cancer					Intervention				Oncological endpoints: 1 = primary, 2 = non‐primary, X = no data reported, XN = no numerical data reported, underline = basis for pre‐trial power analysis, parentheses = major outcomes OS = overall survival, PFS = PFS, RR = response rate, TC = treatment completion, AE = adverse events, Green filling = significant differences reported					
Year	First author	Design	Quality	Pat.N	Tumour type	Stage	Treatment setting^a^	Anti‐cancer treatment^b^	Weight loss at inclusion	Type	Treatment	Control	Duration (week)^c^	Primary endpoints^d^	OS	PFS	RR	TC	AE
1993	Loprinzi [19]	RCT	8	342	Lung 38%, GI 33%	III–IV	2, 3	r, m	> 2.5 kg in 2 m	PA	Megestrol acetate, 4 doses: 160, 480, 800 and 1280 mg/d	Megestrol acetate 160 mg	alat	BW	2				
1993	Ovesen [20]	RCT	8	105	Lung 39%, ovary 43%, breast 18%	III–IV	2	M		ON	Counsel every 2–4 weeks: 30 kcal and 1 g protein per kg	Usual care	20	BW, QoL	2		2		
1996	Gebbia [21]	RCT	6	122	Lung 61%, head–neck 33%, colorectal 18%	III–IV	3		> 5%	PA	Megestrol acetate 320 mg	Megestrol acetate 160 mg	13	Appetite	2				2
1996	Rowland [22]	RCT	10	243	Lung/SCLC 100%	III–IV	2	M		PA	Megestrol acetate 800 g/d vs. placebo	Placebo	16	OS, BW Appetite	**1**	2	2		2
1996	Simons [23]	RCT	7	206	Lung 55%, GI 23%	III–IV	2, 3	m		PA	Medroxyprogesterone acetate 1000 mg	Placebo	12	(Appetite, BW, QoL)	2				2
1997	Beller [24]	RCT	4	240	GI 44%, lung 20%	III–IV	2, 3	m	> 5%	PA	Megestrol acetate 480 mg vs. 160 mg	Placebo	12	QoL	2				2
1999	Westman [25]	RCT	7	255	Colorectal 32%, lung 29%	III–IV	2	r, m		PA	Megestrol acetate 320 mg	Placebo	12	QoL	2				
2002	Jatoi [26]	RCT	10	469	Lung 44%, GI 30%	III–IV	2, 3	m	> 2.3 kg in 2 m	PA	Megestrol acetate 800 mg, dronabinol 5 mg or both	Dronabinol 5 mg	alat	Appetite BW	2				
2002	Ulutin [27]	RCT	9	119	Lung/NSCLC 100%	IV	3		> 10% in 6 m	PA	Megestrol acetate 320 mg	Megestrol acetate 160 mg	12	Appetite BW	2				2
2002	Persson [28]	RCT	6	137	GI 100%	I–IV	1, 2, 3	s, r, m		ON	Counsel every 1–6 m	Usual care	96	(BW)	2				
2003	Bruera [29]	RCT	7	91	GI 35%, lung 18%	III–IV	2, 3	r, m	> 5%	ON	Fish oil 18 g/d	Placebo 18 g/d	2	Appetite	2 XN				2
2004	Jatoi [30]	RCT	8	421	Lung 28%, GI 24%	III–IV	2,3	m	> 2.3 kg in 2 m	ON	ONS‐EPA 2.2 g + megestrol acetate 600 mg per day	ONS‐EPA + placebo or ONS‐Std + MA	12	BW	2				2
2004	Lundholm [31]	RCT	5	309	GI 92%	III–IV	3		3%–5% loss in 3 m	ON, AN	Escalating nutritional care incl. HPN	Usual care	alat	Food intake, BC, REE Exercise capacity	2				
2005	Gordon [32]	RCT	10	50	Pancreatic 100%	III–IV	3		> 10% in 6 m	PA	Thalidomide 200 mg/d	Placebo	24	BW NS	2				
2005	Ravasco [33]	RCT	7	75	Head–neck 100%	I–IV	1	R		ON	Counselling + ONS	Usual care	7	Food intake, BW, QoL					2
2006	Fearon [34]	RCT	8	518	Lung 45%, GI 54%	III–IV	3		> 5%	ON	Eicosapentaenoic acid 2 g/d vs. 4 g/d	Placebo	8	BW	2				
2007	Jatoi [35]	RCT	7	63	Lung 43%, GI 22%	III–IV	2,3	m	> 2.3 kg in 2 m	PA	Etanercept 25 mg sc 2/week	Placebo sc 2/week	24	BW	2				
2008	Wiedenmann [36]	RCT	7	89	Pancreas 100%	II–IV	2	M	> 10% OR > 5% in 3 m	PA	Infliximab 5 mg/kg vs. 3 mg/kg iv every 4 weeks	Placebo	24	LBM	2	2			2
2010	Jatoi [37]	RCT	7	61	Lung/NSCLC, 100%	III–IV	2	M		PA	Infliximab iv 5 mg/kg every 4 weeks	Placebo	8	BW	2	2 XN	2		2
2009	Beijer [38]	RCT	8	100	Lung 45%, GI 27%	III–IV	3	m	> 5% in 6 M	PA	ATP iv: 50 μg/kg/min × 8–10 h per week	Usual care	8	(BW, NS, OS)	2				
2011	Baldwin [39]	RCT	8	358	GI 74%, lung/NSCLC 23%	III–IV	3	M	> 0% in 3 m	ON	Counselling 600 kcal/d +/− ONS 588 kcal/d	Usual care	6	OS	**1**				
2012	Kraft [40]	RCT	10	72	Pancreatic 100%	IV	2, 3	m		PA	Carnitine 4 g/d per os	Placebo	12	TNF‐α	2				
2012	Madeddu [41]	RCT	7	60	Head–neck 23%, lung 21%, GI 12%	III–IV	2, 3	m	> 5% in 6 m	PA	Megestrol acetate 320 mg + celecoxib 300 mg + carnitine 4 g	Celecoxib 300 mg + carnitine 4 g	16	LBM, Physical activity	2	2	2		2
2012	Silander [42]	RCT	6	134	Head–neck 100%	III–IV	1	s, R, m		ON, AN	Counsel and prophylactic PEG tube: 30 kcal and 1.2–1.5 g protein/kg for 1–12 m	usual care	104	(BW, QoL, length of hospital stay)	2	2		2	2
2013	Del Fabbro [43]	RCT	10	73	GI 56%, lung 44%	III–IV	2, 3	m	> 5% in 6 m	PA	Melatonin 20 mg/d	Placebo	4	Appetite	2				
2014	Bourdel‐Marchasson [44]	RCT	10	341	GI 39%, lymphoma 15%, lung 10%	III–IV	2	M		ON	Counsel ad 30 kcal/kg, protein 1.2 g/kg	Usual care	16	OS	**1**		2	2	2
2014	Pottel [45]	RCT	8	85	Head–neck 100%	I–IV	1	R, m		ON	Echium oil (n‐3 rich) 15 mL/d	Sunflower oil 15 mL/d	7	BW					2
2016	Coats [46]	RCT	10	87	Lung/NSCLC 67%, colorectal 33%	III–IV	2, 3	m	> 5%	PA	Espindolol 10 mg/d vs. 2.5 mg/d	Placebo	16	BW	2				2
2016	Jatoi [47]	RCT	8	118	Lung 37%, GI 26%	III–IV	2, 3	r, m	> 2.3 kg in 2 m	ON	White wine twice per day	ONS of choice	4	Appetite	2				2
2016	Temel [48]	RCT	8	979	Lung/NSCLC 100%	III–IV	2, 3	r, m	> 55 in 6 m	PA	Anamorelin 100 mg/d (67% of patients)	Placebo (33% of patients)	12	HGS LBM	2				
2016	Woo [49]	RCT	9	67	Pancreatic 100%	III–IV	2, 3	m		PA	Pancreatin 6–9 × 458 mg/d	Placebo	8	BW	2				2
2017	Jatoi [50]	RCT	8	263	Lung 36%, GI 26%	III‐IV	2, 3	m		PA	Creatine 2 g/d	Placebo	alat	BW	2				2
2017	Leedo [51]	RCT	8	40	Lung 100%	II–IIV	1, 2	s, r, m		ON	Home meal service	Usual care	12	QoL	2				2
2017	Solheim [52]	RCT	8	46	Lung/NSCLC 57%, pancreas 43%	III–IV	2	M	< 20% loss in 6 M	PA, ON	ONS 440 mL, EPA 2 g, celecoxib 300 mg, exercise 5/week	Usual care	6	Feasibility, compliance	2				2
2017	Zietarska [53]	RCT	6	95	Colorectal 100%	II–IV	1, 2	M	< 10% loss	ON	ONS protein‐rich 2 × 300 kcal vs. usual care	Usual care	12	(Toxicity)				**1**	**1**
2018	Britton [54]	Cluster‐RCT	8	307	Head–neck 100%	I–IV	1	R, m		ON	Counselling	Usual care	17	NS				2	2
2018	Golan [55]	RCT	7	125	Pancreatic 100%	II–IV	2	M		PA	Antimyostatin LY2495655 iv, 300 mg or 100 mg every 2 weeks	Placebo	alat	OS	**1**	2	2		2
2018	Katakami [56]	RCT	8	174	Lung/NSCLC 100%	III–IV	2, 3	r, m	> 5% in 6 m	PA	Anamorelin 100 mg/d	Placebo	12	LBM	2		2		2
2018	Uster [57]	RCT	9	58	GI 66%, lung 34%	III–IV	2, 3	M		ON	3 × counsel + exercise training	Usual care	12	QoL	2				2
2018	Xie [58]	RCT	8	54	Lung/NSCLC, 100%	IV	3		5%–10% loss	PA	Thalidomide 150 mg/d vs. usual care	Usual care	12	(NS, QoL, AE, OS)	2				2
2019	Akita [59]	RCT	8	62	Pancreatic 100%	II	1	R, M		ON	ONS (560 kcal, EPA‐enriched, Prosure)	Usual care	5	Muscle mass					2
2019	Cereda [60]	RCT	8	166	Lung 26%, GI 54%	III‐IV	2	M	> 10% in 6 m	ON	Counselling (ONS, EN/PN if req.) + whey protein suppl. 20 g/d	Counselling (ONS, EN/PN if req.)	12	Bioimpedance phase angle					2
2019	Laviano [61]	RCT	8	56	Lung/NSCLC, 100%	I–IV	2	M	< 11% loss	ON	ONS 400 kcal: (whey + N‐3 2 g + Vit D 10 μg)	ONS standard	12	AE	2		2	2	**1**
2019	Obling [62]	RCT	7	47	GI 100%	III‐IV	2, 3	m		ON, AN	SPN: 9 kcal/kg, protein 0.5 g/kg	Usual care	24	Fat‐free mass	2				
2020	Bouleuc [63]	RCT	7	111	GI 29%, lung 19%	III–IV	2, 3	m	2%/w, 5%/m, 10%/6 m	AN	Supplemental PN: 1000 kcal/d −35 kcal/kg/d	Usual care	alat	HR‐QoL	2				2
2020	Huang [64]	RCT	7	114	Nasopharyngeal 100%	III–IV	1	R, M		ON	ONS to reach 30 kcal/kg	Usual care	alat	OS	**1** X			2	2
2020	Qiu [65]	RCT	6	96	Oesophageal 100%	II–III	1	R, M	No severe malnutr	ON	Counselling + ONS ad > 25 kcal and 1.2 g protein/kg	Usual care	5	AE			2		**1**
2020	Storck [66]	RCT	10	52	Lung 42%, GI 27%	III–IV	2,3	m		ON	ONS‐whey‐Leu 21 g/d + counsel +exerc training 3/week	Usual care	12	SPPB	2 XN				2
2020	van der Werf [67]	RCT	9	107	GI 100%	IV	2	M		ON	Nutritional counselling	Usual care	12	Muscle mass	2	2		2	2
2021	Bargetzi [68]	RCT	8	506	Lung 22%, GI 17%, haematologic 21%	I–IV	1, 2, 3	r, m		ON	Counsel, ONS, PN: 30 kcal/kg	No nutritional care	alat	OS	**1**				2
2021	Hunter [69]	RCT	7	120	GI 30%, lung 11%	III–IV	2, 3	m	> 5% in 6 m	PA	Mirtazapine 15 mg	Placebo	8	Appetite	2				2
2021	Izumi [70]	RCT	6	81	Urologic 63%, GI 15%, lung 12%	III–IV	2	M		PA	Testosterone enanthate 250 mg im every 4 weeks	Usual care	8	QoL	2				2
2021	Kita [71]	RCT	6	87	Oesophageal 100%	IIA–IV	1	M		AN	EN/ONS 600 kcal/d	PN 600 kcal/d	2,5	Muscle mass				2	2
2021	Kutz [72]	RCT	7	58	Head–neck	I–IV	1	R, m		ON	Counselling every 2 weeks	Usual care	alat	QoL	2				2
2021	Meng [73]	RCT	8	353	GI 100%	I–IV	1	S, m		ON	Counselling + ONS	Counselling	12	BW Muscle mass				2	2
2023	Almeida [74]	RCT	8	52	GI 73%, lung 17%, urologic 6%	II–IV	2	R, m		PA	Mirtazapine 30 mg/d	Megestrol 320 mg/d	8	BW				2	2
2023	Sandhya [75]	RCT	9	124	GI 65%, lung 35%	III–IV	2	M		PA	Olanzapine 2.5 mg/d	Placebo	12	BW Appetite				2	2

Abbreviations: alat = as long as tolerated, AN = enteral or parenteral nutrition, BC = body composition, BW = body weight, HGS = hand grip strength, HR‐QoL = health‐related quality of life deterioration‐free survival, LBM = lean body mass, NA = not applicable, NS = nutritional status, ON = oral nutrition, PA = pharmacologic agent, QoL = quality of life, SPPB = short physical performance battery.

^a^ 1: curative; 2: palliative anti‐cancer treatment; 3: best supportive care without anti‐cancer treatment.

^b^ s, S: surgery; r, R: radiotherapy; m, M: systemic treatment; s, r, m: some patients; S, R, M: all patients.

^c^ Reported duration of response as an additional secondary endpoint.

^d^ Endpoints used to perform sample size calculations are underlined; if no explicit primary endpoint was designated, ‘major outcomes’ as presented in the abstract of the trial are given in brackets.

In summary, 27 trials (47%) were performed in a single tumour site: lung cancer (ten studies), pancreatic cancer (six studies), head and neck cancers (six studies), oesophageal and gastric cancers (three studies) and colorectal cancers (two studies). Thirty studies included multiple tumour types. Overall, 39 studies (68%) included patients with lung cancer, and 39 studies included patients with gastrointestinal (GI) cancers. Forty‐two studies (74%) included patients with Stage III–IV disease. Of the 57 studies, nine included only curative settings (16%), five included both curative and palliative settings (9%), 13 included patients receiving palliative anti‐cancer treatments (23%), 24 trials investigated both patients receiving palliative anti‐cancer treatments or best supportive care only (42%), and six trials included only patients receiving best supportive care without any anti‐cancer therapies (10%). Forty‐nine per cent of studies included a pharmacological intervention, 49% included an oral nutrition intervention and 9% an enteral or parenteral nutrition intervention with four trials (7%) offering a combination of oral plus either enteral/parenteral or a pharmacological intervention.

**FIGURE 2:**
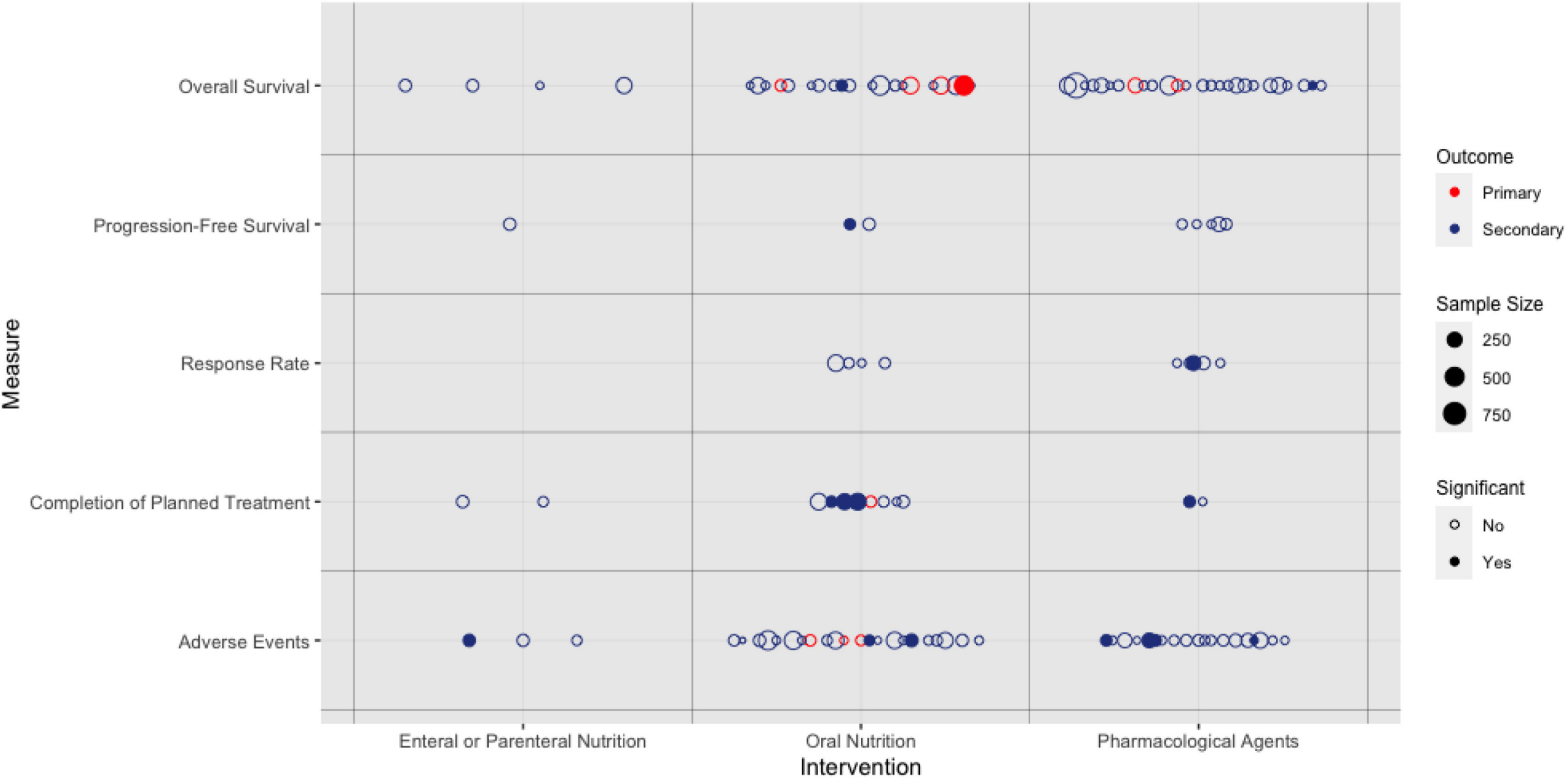
The relationship between interventions and oncology endpoints in cachexia clinical trials.

Figure 2 shows a balloon plot showing the relationship between study interventions, endpoints used, sample size and statistical significance. Six different endpoints were assessed across the 57 studies: OS, PFS, RR, completion of treatment, AE and duration of response. Only a single study had duration of response as an endpoint [55] but also reported OS, PFS, RR and AE. One study reported health‐related QoL deterioration‐free survival, but this was considered to be a QoL endpoint, not an oncological one [63], and was not included here but in the QoL review of this endpoint series [2].

For each trial, the primary endpoint(s) or if none could be determined the ‘major outcomes’ are listed in Table 1. Primary endpoints, which served to perform sample size calculations, are designated by underlining, and major outcomes are placed in parentheses. Table 1 also lists all oncological endpoints reported in each study and whether they were studied as primary (‘1’) or non‐primary endpoints (‘2’).

The most common outcome was OS (46/57 trials, 80%), but it was the primary outcome for only six studies. Three studies (one as a primary endpoint) reported a significant positive effect on survival [58, 67, 68]. AEs were reported in 42/57 (74%) of trials, three using it as a primary endpoint; seven trials presented significant differences between study arms, but only one as a primary endpoint [22, 60, 63, 65, 69, 74, 75]. Out of 27 trials studying other endpoints such as PFS, RR, completion of treatment and duration of response, six were positive: one for PFS, one for RR and four for completion of treatment, but none as a primary endpoint.

There were four studies, which intended to report an oncology endpoint, but either did not present the data or failed to provide numerical data for this endpoint [46, 47, 64, 66,29,37]. These studies are highlighted in Table 1.

The 57 trials assessed a total of 115 oncological outcomes, and of these, 13 trials reported 16 positive outcomes. Only two studies found a primary endpoint (OS or AE) to be positive [65, 68]. Fifteen positive endpoints were observed in studies involving at least some patients receiving anti‐cancer drug treatment, and 13 occurred in trials in a palliative setting. In terms of interventions, seven positive endpoints were in the context of a trial investigating a drug, eight oral nutritional interventions and one enteral/parenteral nutrition.

Among the 13 studies reporting positive oncological outcomes, nine [22, 54, 58, 60, 65, 68, 73, 74, 75] had positive primary outcomes. However, only two studies, investigating comprehensive nutritional support, reported primary oncological endpoints, OS [68] or AE [65], respectively. The remaining seven studies were heterogeneous, with three [54, 60, 73] focusing on nutritional interventions—each with different primary non‐oncological outcomes. The other four [22, 58, 74, 75] studies investigated separate pharmacological interventions. Due to this variability, drawing any robust conclusion on interactions between oncological endpoints and other categories of outcomes is not possible.

### 3.1 OS

The most frequent endpoint reported was OS in 46 of 57 studies (80%) (Figure 2). Three trials were performed in a curative and 43 in a palliative setting. In 38 studies, anti‐cancer treatments were given, but in eight studies, patients received best supportive care only. Sixteen trials explored interventions with oral nutrients, four with tube feeding or parenteral nutrition and 26 studies with pharmacologic agents.

Of six studies using OS as the primary endpoint [22, 39, 44, 55, 64, 68], four had the trial intervention given concurrently with palliative systemic anti‐cancer therapy (SACT) [22, 39, 44, 68,55]. One study involved patients receiving a curative treatment and one study involved mainly patients receiving best supportive care. Four trials explored nutritional interventions. Of these six trials stating that OS was the primary endpoint, one did not report these results [64], and one trial reported a positive finding [68]. Of the 40 trials with OS as a secondary endpoints, two trials showed improved survival in the intervention arm [58, 67], and two trials did not report numerical data [29,^66^].

**FIGURE 3:**
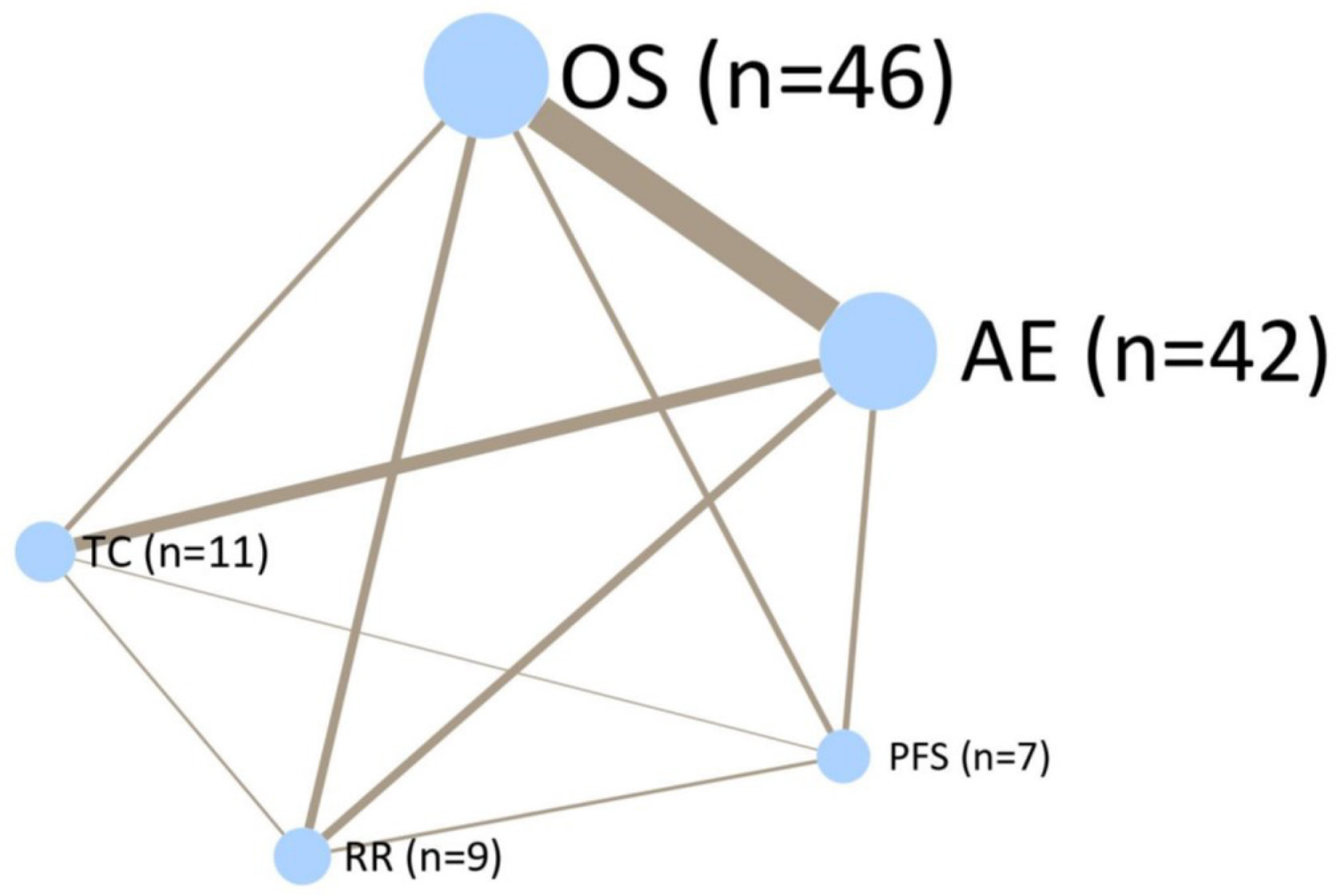
Network diagram of the relationship between oncology endpoints in cachexia trials. AE = adverse event, OS = overall survival, PFS = progression‐free survival, RR = response rate, TC = completion of treatment.

**FIGURE 4:**
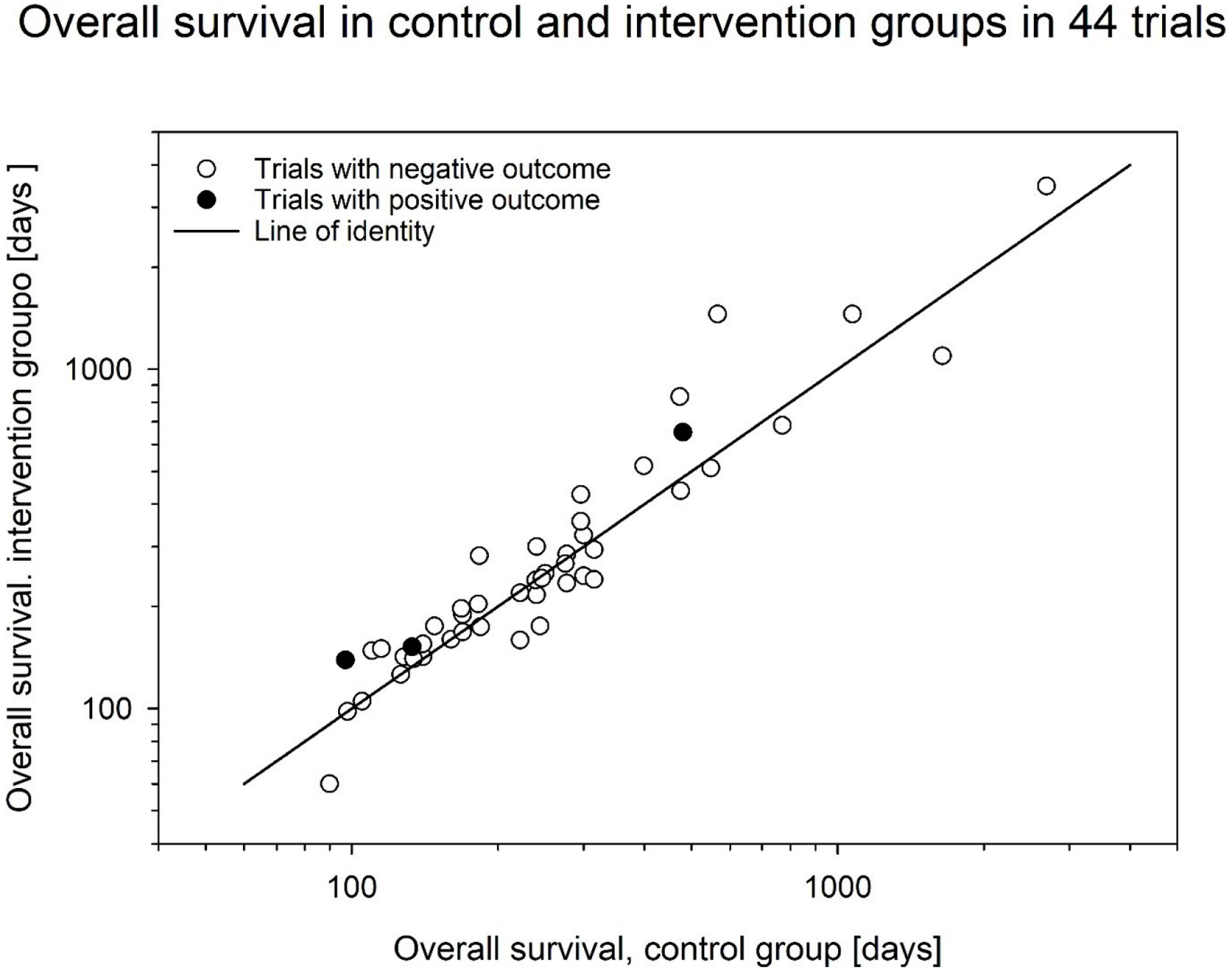
OS in control and intervention groups of each trial. Of 46 trials targeting OS, two did not report results [46, 75]. Five trials included two interventions groups [18, 27, 42, 47, 73], allowing each group to be compared to the control group. One trial reported 30‐day as well as 6‐month survival for the active and control groups, allowing both comparisons to be plotted [63]. Three trials with positive outcomes are plotted in black [63, 67, 68]: from lowest to highest OS.

Figure 3 shows a network plot demonstrating how different endpoints are related between different trials. OS was assessed in 46 trials, and of these, 30 also assessed AEs, which were assessed in 42 studies in total. Figure 4 shows the relationship between OS in the intervention arms versus control arms in the eligible trials. Consistent with the large heterogeneity in disease settings, trials differed widely with respect to median survival of the included patients with data ranging from 60 to 3468 days and were scattered around the line of identity.

Of the studies using OS as a primary endpoint, three trials reported pre‐trial sample size calculations targeting HR of 0.67 [22], 0.76 [39] and 0.74 [44]. Two of these trials were stopped prematurely after recruiting 42% [44] or 54% [39] of the planned patients due to the recommendation of a data review committee [39] or an insufficient rate of inclusion [44], but Rowland et al. recruited 97% of the planned sample size [22]. Golan et al. studied the effect of a myostatin‐neutralizing antibody [55]. They did not report a pre‐trial power analysis but also ended their trial prematurely due to the decision of an internal assessment committee after noting an imbalance in overall survival during follow‐up in one of two intervention groups [55]; with the sample size reached, the authors reported a power of 0.74 with a type I error of 0.20 to detect HR of 0.83 and 0.71 in the low‐dose and high‐dose arms, respectively, of their trial. Finally, Bargetzi et al. reported a secondary analysis of data collected during the previously published EFFORT trial [76] but without providing a power analysis [68].

Three of the 46 trials targeting OS reported significant differences in survival. Bargetzi et al., in a secondary analysis of patients with cancer recruited to the EFFORT trial [76], targeted 30‐day mortality as the primary endpoint. This trial compared escalating individualized nutritional support versus standard care in 506 patients with different tumours in either curative or palliative settings. Within 30 days, in the control group, 50 patients (19.9%) died compared to 36 (14.1%) in the intervention group, giving an adjusted odds ratio of 0.57 (95% CI 0.35–0.94; *p* = 0.027). There was no difference between groups in 180‐day mortality, and no reference to multiple secondary analyses published separately from the same data set was made.

Two smaller trials targeting OS as a secondary endpoint also reported significant differences between treatment groups. Xie et al. [58] compared the effect of thalidomide (150 mg/day for 12 weeks) in 54 patients with non–small cell lung cancer (NSCLC) who also received an Asiatic toad extract (cinobufagin). Patients included had established cachexia and were not receiving anti‐cancer treatment. After a follow‐up of more than 200 days, OS was higher in the thalidomide group (59 vs. 30%, *p* = 0.011), corresponding to a median survival of 266 versus 113 days and an impressive HR of 0.42 [58]. Van der Werf et al. randomized 107 patients with metastatic colorectal cancer receiving first‐line chemotherapy and compared repeated dietary counselling to usual care [67]. The intervention resulted in significantly longer PFS (9.6 vs. 7.6 months, HR = 0.79, *p* = 0.039) and OS (21.7 vs. 16.0 months, HR = 0.74, *p* = 0.046). The results of both trials [58, 67], however, were compromised by multiple testing of further secondary endpoints.

Four studies did not find a difference in OS as a primary endpoint. In a mixed group of 358 patients with advanced GI or lung cancers, Baldwin et al. [39] reported no differences in 1‐year survival after 6 weeks of either nutritional counselling, supplying oral nutritional supplements (ONS) with low compliance or standard of care. Bourdel‐Marchasson et al. [44] compared 16 weeks of nutritional counselling to standard of care in 341 patients with advanced cancer or lymphoma and found no difference in 1‐year survival. Rowland et al. [22] explored the role of megestrol acetate versus placebo given for 16 weeks to 243 patients with small cell lung cancer but observed no effect on 1 year survival. Finally, Golan et al. [55] investigated the anti‐myostatin antibody LY2495655 in 125 patients with Stage II–IV pancreatic cancer given in two different doses (100 and 300 mg) compared to standard treatment alone without an effect on OS. Of note, although deaths on study drug and within 30 days of discontinuation were balanced across treatment arms, the study was discontinued when an interim analysis detected an imbalance in deaths among patients with long‐term follow‐up. The highest death rate occurred in patients receiving the 300 mg dose [18].

### 3.2 PFS and Duration of Response

*PFS* is defined as the time from randomization to disease progression or death from any cause. Seven studies assessed PFS, none as a primary endpoint [37,^22^, ^36^, ^41^, ^42^, ^55^, ^67^]. One of these studies also assessed duration of response [55]; duration of response is PFS restricted to patients reaching a complete or partial remission. One trial was performed in a curative setting and six in a palliative setting. In all seven trials, anti‐cancer treatments were given. Two trials explored an intervention with nutritional counselling [42, 67] with one of them studying counselling in combination with prophylactic placement of an enteral feeding tube in patients undergoing definitive radiotherapy or chemoradiotherapy for head and neck cancer [42]. The other five trials investigated pharmacological agents including megestrol acetate [22, 41], infliximab [36, 37] and the anti‐myostatin antibody LY2495655 [55].

Only Van der Werf et al. [67] reported a positive effect on PFS (*p* = 0.039). Their study compared the effect of nutritional counselling including optional ONS with usual care in patients with metastatic colorectal cancer undergoing first‐line chemotherapy [67]. In addition to the primary endpoint of muscle mass, they reported several secondary endpoints including PFS, OS, treatment intensity and AE but did not adjust their statistical analysis for multiple testing.

### 3.3 RR

Nine studies reported RR, none as a primary endpoint [20, 22, 37, 41, 44, 55, 56, 61, 67,65]. One trial was performed in a curative setting [65] and eight in a palliative setting. In eight of these trials, anti‐cancer treatments were given to all patients, whereas one was performed in some patients receiving best supportive care only [41]. Four trials explored interventions with oral nutrients [20, 44, 61, 67,65], and five investigated pharmacological agents including megestrol acetate [22, 41], infliximab [37], anamorelin [56] and the anti‐myostatin antibody LY2495655 [55].

Only one of these studies reported a statistically significant difference in RR, and this was an unexpected significantly poorer outcome in the investigational arm [22]. The study compared the role of megestrol acetate to placebo in patients receiving chemotherapy for extensive‐stage small cell lung cancer. Primary outcome parameters were QoL and OS, secondary outcome parameters included, but were not limited to RR, PFS, AE and body weight. In the megestrol acetate cohort, RR was worse (68% vs. 80%, *p* = 0.03); there was a trend towards poorer OS (8.2 vs. 10.0 months, *p* = 0.49), and a significantly higher rate of thromboembolic events was noted (9% v 2%, *p* = 0.01). There were no statistically significant differences in baseline patient characteristics. No adjustments were made for multiple testing.

### 3.4 Therapy Interruption/Completion of Treatment

Eleven studies reported completion of treatment (TC) as an endpoint. Treatment completion rates included rates of treatment delays, studied in five trials 42, 53, 54, 61, 64, 71, 73, 74,44], dose reductions studied in nine trials [44,53,61,64,67,71,73‐75] and treatment interruptions in six trials [42,53,54,67,73,74]. One study targeted completion of treatment as a primary endpoint [53], but the other remaining 10 trials used completion of treatment as a secondary endpoint [42, 44, 54, 61, 64, 67, 71, 73, 74, 75]. Four trials were performed in a curative setting [42, 54, 64, 71] and seven in a palliative setting. In all trials, anti‐cancer treatments were given. Seven trials explored interventions with oral nutrients [42, 44, 53, 54, 61, 64, 67, 73], two with feeding tubes [42, 71], and two trials investigated pharmacologic agents, either mirtazapine [74] or olanzapine [75].

The only study using completion of treatment as a primary endpoint reported dose reductions, treatment delays as well as interruptions but observed no differences between groups [53]. Four of the other trials reported significant differences between study groups. Britton et al. [54] compared standard nutritional counselling by dietitians to counselling performed by specially educated oncology dietitians in 307 patients with head and neck cancers undergoing curative radiotherapy. The primary outcome of the trial was nutritional status, and several secondary endpoints were monitored. They observed a significant reduction in radiotherapy interruptions (*p* = 0.04); however, there was no adjustment for multiple testing. Huang et al. [64] compared offering prophylactic ONS to usual care in 114 patients with head and neck cancers undergoing curative chemoradiotherapy. The primary outcome was OS, and several secondary endpoints were monitored. The authors observed a significant reduction in treatment interruptions (*p* = 0.04); however, there was no adjustment for multiple testing. Meng et al. compared nutritional counselling without offering ONS to counselling plus ONS in 353 patients after gastrectomy for gastric cancer and at risk of malnutrition [73]. The primary outcomes were body weight and muscle mass, and several secondary endpoints were reported. The authors observed a highly significant reduction in the fraction of patients requiring either chemotherapy dose reductions, treatment delays or treatment interruptions (*p* = 0.004); however, unfortunately, the authors did not report whether the study groups differed with respect to the fraction of palliative versus curative settings and the fraction of patients receiving chemotherapy. Finally, Sandhya et al. [75] compared the effect of low‐dose olanzapine versus placebo in 124 newly diagnosed patients with advanced GI or lung cancers receiving palliative chemotherapy. The primary outcomes of appetite and body weight, both improved in the olanzapine group compared to the placebo group. Of several secondary endpoints reported, the authors observed a highly significant decrease in the occurrence of chemotherapy dose reductions (*p* < 0.001). From their results, the authors suggested that olanzapine may be considered an add‐on therapy in the studied patient category.

### 3.5 Treatment Toxicity and AEs

In contrast to other outcomes, toxicities and AEs reported in cancer cachexia trials comprise a more heterogeneous group of different outcomes referring to any or a selection of untoward effects occurring during the clinical trial. Thus, a positive finding could be one of a larger group of parameters tested. Due to the often‐large number of reported parameters, statistical analysis would require adaptation to multiple testing, which usually was lacking in the analysed trials.

Treatment toxicity and AEs were investigated as an endpoint in 42 studies. AEs may be induced by anti‐cachexia interventions or if induced by anti‐cancer treatment may be alleviated or prevented by an anti‐cachexia intervention. Three of the 42 studies used AEs as a primary endpoint [53, 61, 65]. One of these trials monitored AEs induced by the anti‐cachexia intervention [61]; the other two studies aimed to decrease radio‐ or chemotherapy‐induced AEs by an anti‐cachexia intervention [53, 65].

Two studies targeting AE as a primary endpoint were negative [53,^61^], whereas one study reported a positive effect, albeit without adjusting for extensive multiple testing [65]. Zietarska et al. [53] compared ONS versus usual care in 114 patients with colorectal cancer receiving chemotherapy. Patients differed in tumour stages II–IV, with 32% having Stage IV disease. AE were recorded every 4 weeks and graded according to CTCAE v 4.0; no significant effects were detected. Laviano et al. [61] compared a whey protein–based ONS enriched in fish oil and 25‐hydroxyvitamin D3 to a standard ONS in 55 patients with advanced NSCLC receiving first‐line chemotherapy. The primary outcome were AE occurring during the study as assessed every 3 weeks. Assessment and grading of AE were not further specified, and no statistical analysis was performed. Qiu et al. [65] randomized 96 patients with newly diagnosed oesophageal cancer undergoing definitive chemo‐radiotherapy to a personalized nutrition regime including ONS versus usual care. The primary outcome was AE as defined by the American Radiation Therapy Collaboration. The more aggressive nutrition approach led to a significant decrease in two of several monitored AE, namely, a reduction in radiation oesophagitis (*p* = 0.029) and skin toxicity (*p* = 0.015), but no effects on myelosuppression and rate of infections. The true number of primary endpoints monitored is not given, and no adjustment for multiple testing is reported.

Of the 39 studies assessing AE as a secondary endpoint, six studies showed a significant difference in at least one AE (*p* < 0.05). Thirty‐three studies had only negative secondary AE outcomes.

Of the six studies reporting a significant effect on AE, four used a pharmacological intervention [22, 69, 74, 75], and two used nutritional interventions [60, 63]. Studies by Almeida et al. [74] and Sandhya et al. [75] reported G3/severe toxicities as a single value, whereas the other four studies reported occurring AEs separately. None of these mentioned adjusting for multiple testing, such as the inclusion of a Bonferroni correction [22, 60, 63, 69].

### 3.6 Compliance

Compliance with the study protocol was assessed in the 28 trials investigating oral nutritional interventions. Of these, 13 studied nutritional counselling, and 15 studied an oral nutritional product (e.g., ONS and capsule). Compliance of the study investigators with providing the scheduled intervention was reported in only four of the studies on nutritional interventions. Patient compliance was reported by 24 of the 28 studies (86%). Fractional patient compliance was reported by 16 studies, including 14 of the 15 studies of an oral nutritional product (median 69%, IQR 54 to 77%). Compliance‐associated biomarkers were reported by two studies.

### 3.7 Comparison of Oncological to Traditional Cachexia Endpoints

To compare the outcome of oncological to more traditional cachexia endpoints, we queried the endpoints monitored in the three trials, which reported significant differences in OS, the most frequently studied oncological endpoint [58^63^, ^67^, ^68^]. Unfortunately, potential inferences are weakened by the facts that none of these studies was blinded and none provided statistical adjustment for multiple testing. Bargetzi et al. studied 30‐day survival as a primary endpoint in 506 patients with different cancer types and stages comparing escalating care by a dedicated nutritional support team to usual care without contact with a nutrition team [68]. Thirty‐day survival was higher in the intervention group (*p* = 0.027) as were two of eight secondary outcomes, QoL assessed by the European Quality of Life 5 Dimensions Index (*p* = 0.021) and functional outcome (*p* = 0.021). Van der Werf et al. studied 107 patients with metastasized colorectal cancers undergoing first‐line chemotherapy and compared dedicated dietetic counselling to usual care during up to 6 months of treatment [67]. OS and PFS were among 46 reported outcome measures; both OS (*p* = 0.046) and PFS (*p* = 0.039) were higher in the intervention group, as was the change in body weight after two cycles (*p* = 0.045) but not four cycles of chemotherapy. Xie et al. studied 54 patients with advanced cancer receiving best supportive care and the toad extract cinobufagin [58]. The intervention group was treated with thalidomide for 12 weeks, whereas the control group remained untreated. In addition to OS and AE, several further outcome parameters were compared at two different time points, resulting in more than 30 statistical tests. In this unblinded study, OS was higher in the thalidomide group (*p* = 0.011), as were the change in body weight (*p* = 0.031), serum albumin (p = 0.01), handgrip strength (*p* < 0.001) and QoL assessed with the EORTC QLQ‐C30 tool (*p* < 0.001).

#### 3.7.1 Statistical Considerations

Clinical trials usually are powered to detect postulated differences between study groups in the primary outcome(s), and an appropriate pre‐trial power analysis should be reported [18]. Low statistical power will result in missing true effects and at the same time increasing the ratio of false (Type I error) versus true positive findings [77, 78]. However, few CC trials studied oncological outcomes as primary endpoints [22, 39, 44, 53, 55, 61, 64, 65, 68], and in less than half of these, a pre‐trial sample size analysis was reported [22, 39, 44, 65]. Of these, two trials were terminated early [39, 44], and only two trials recruited the projected number of participants [22, 65].

Most trials analysed in this review observed no statistically significant changes in oncological outcomes, but the few findings reported as significant may be unreliable based on the lack of adjustment for multiple testing. Statistical testing for several separate outcomes requires adequate adaptation of the testing level to avoid increasing the probability of a Type I error and falsely rejecting the null hypothesis. Without such adaptation, the results of secondary outcomes and multiple testing may be considered only exploratory. Of the two studies reporting positive primary oncological outcomes, Qiu et al. reported improvements by comprehensive nutrition support in radiation‐induced skin toxicity and esophagitis at *p* levels of 0.015 and 0.029, respectively. However, they reported statistical comparisons on more than 20 further outcomes without referring to multiple testing [65]. Similarly, Bargetzi et al. reported a positive effect of comprehensive nutritional support on 30‐day mortality at a level of *p* = 0.027 [68] in a secondary analysis of a larger trial published previously [76]. Currently, more than 15 secondary analyses of the same original data set have been published without presenting a procedure for adjusting the *p* level for such multiple testing. Studies reporting significant findings in non‐primary oncological outcome, reported at least four [74] and in some cases more than twenty [64] or more than thirty further outcomes [58], each without referring to multiple testing.

## 4 Discussion

This review identified 57 randomized cancer cachexia clinical trials reporting oncological endpoints. OS was reported most frequently by 46 of the 57 trials, AE by 42 trials, but other endpoints including completion of treatment, RR and PFS were studied less often, by 11 or fewer trials. A major challenge in conducting this review and drawing meaningful conclusions was the significant heterogeneity among the eligible trials. This variability spanned across treatment intent (curative vs. palliative), cancer therapy type and efficacy, cachexia interventions and the potential interaction between cancer therapy and cachexia outcomes. Further, some of the populations studied may have had refractory cachexia and/or were dying from general debility. As a result, any observed effects on survival or other oncological endpoints, whether positive or negative, must be interpreted with caution.

Oncological endpoints are used to assess the benefits and harms of cancer treatments and encompass a variety of measures accepted in oncological trials. These endpoints include measurements of OS, evaluation of tumour response (e.g., objective RR, pathological complete response and clinical benefit rate) and duration of tumour control (e.g., PFS, time to progression, disease‐free survival and median survival). They also cover treatment completion and AE. However, not all of these endpoints were reported in the trials included in this review. Some oncological endpoints are accepted as clinical endpoints, whereas others are surrogate endpoints. Clinical endpoints represent direct clinical benefit and should generally take precedence over surrogate endpoints when planning a cancer cachexia trial. Additionally, not every endpoint is relevant in all stages of cancer, with different choices potentially better suited to patients in the neoadjuvant, curative, early or late palliative settings. Guidance from the Food and Drug Administration (FDA), echoed by others, supports that oncological endpoints include OS, disease‐free survival, response evaluation and patient reported outcomes, among others [7, 79].

OS is a key cachexia endpoint, but it has also been accepted as a ‘gold standard’ for primary endpoints in oncological trials and is a key endpoint in oncology clinical trials supported by both the FDA [80], EMA [81] and others [7]. However, especially in curative settings, it may require long follow‐up; if OS is longer than an intervention, other factors including subsequent treatments may dilute an expected effect [82]. OS may be a reasonable endpoint for investigating anti‐cachexia treatments that aim to support body composition and metabolism. By directly enhancing resilience and survival, and indirectly improving outcomes through increased tolerance to and effectiveness of anti‐cancer therapies, OS becomes a valuable measure. Trials involving subjects with advanced cancer and an expected survival of less than 1 year might be well suited to employ OS as a primary or secondary endpoint, provided that the sample size is appropriately adjusted. In this review, OS was an endpoint in 46 of the 57 trials analysed, yet it was chosen as a primary endpoint in only six trials.

As malnutrition may be a relevant factor impairing response to immuno‐oncology therapies [83, 84], it appears especially interesting to study anti‐cachexia treatments in these settings. Thus, if OS were deemed an appropriate endpoint for cancer cachexia trials, reducing sample size will require either increasing the effect sizes by selecting more potent interventions or decidedly reducing the heterogeneity among the study participants. Important options to reduce heterogeneity would be to limit trial participation to one or few cancer entities, disease stages and treatment settings with similar prognosis.

PFS is a surrogate endpoint primarily used in advanced and metastatic cancer settings. It offers the advantage of requiring a much shorter follow‐up period and is not confounded by subsequent treatments. Although PFS allows patients in oncological trials to switch to the investigational agent upon progression [85], this is not relevant in trials investigating anti‐cachexia interventions. PFS is appropriate only in trials involving anti‐cancer treatments, where its outcome reflects the indirect effects of anti‐cachexia interventions.

Defining progression in the context of PFS is challenging due to the increasing variety of available anti‐cancer treatments and the complexity of treatment responses. For example, immunotherapy requires specific guidelines to assess progression because of the diverse patterns of response and progression [86]. Additionally, PFS is not necessarily a reliable proxy for OS or QoL. A recent large meta‐analysis of 91 oncological studies found that only half supported an association between PFS and OS, with a third of the positive studies lacking data to substantiate their conclusions [87]. Furthermore, a meta‐analysis of 38 randomized clinical trials involving 13 979 patients by Kovic et al. found no association between PFS and QoL [88].

Common sense suggests that anti‐cachexia therapies, like other supportive treatments, would more probably improve tolerance to an anti‐cancer agent rather than enhance its efficacy. Thus, monitoring the completion rates of planned anti‐cancer dosing might be a more appropriate endpoint to study than PFS. On the other hand, endpoints such as PFS could help control for confounding factors that impact cachexia when alternative endpoints are being assessed. Cachexia typically improves with cancer regression and worsens with cancer progression; therefore, the effects of an anti‐cachexia intervention are influenced by the direction and degree of tumour activity. Consequently, a cachexia trial should include the status of the cancer during the intervention to avoid the severe risk of observing effects that are solely due to changes in tumour control. Following this logic, PFS may be a valid option for stratifying responses to an anti‐cachexia intervention.

Objective RR has been accepted as a good measure of anti‐tumour activity. RR should be determined by a rigorous methodology (e.g., RECIST) [89] and is most appropriately used in neoadjuvant settings. RR does have some drawbacks in the oncological setting by not detecting stable disease and not differentiating between complete and partial responses. Similar to PFS, because it is a measure of tumour activity, RR is not an ideal outcome in cachexia trials. However, like PFS, RR may be a tool to stratify results in cachexia trials based on cancer activity during the trial. Nine of 54 studies used RR as a secondary, but none as a primary endpoint. No study used RR for stratification of results. Only one of the 9 studies was performed in a curative setting, and, interestingly, one trial was performed without concurring anti‐cancer treatment.

Monitoring treatment completion rates for planned anti‐cancer therapy helps assess the impact of anti‐cachexia interventions on the tolerability of these treatments. An effective anti‐cachexia intervention should improve, or at least not reduce, the tolerability of anti‐cancer agents, allowing for the completion of planned treatments. This principle applies not only to chemotherapy [90] but also immunotherapy where presence of cachexia has a negative impact on treatment efficacy in both NSCLC [91, 92] and gastrointestinal malignancy [93].

Using completion of treatment as an endpoint is crucial in neoadjuvant and adjuvant cancer treatments, where substandard dosing can decrease RR and OS. We found 11 trials reporting on dose modifications and treatment intervals of anti‐cancer therapies, including three of five trials focused on head and neck cancer. Definitive radio‐ or radiochemotherapy for this disease is complex and toxic, delivered over several weeks. Delays or missed days of radiotherapy can affect outcomes, making timely treatment delivery essential.

The benefit of supportive interventions in cancer patients can be seen in the reduction of harm from anti‐cancer therapies. This is particularly crucial for patients suffering from malnutrition, which causes nutritional and metabolic deficiencies [94, 95, 96]. AEs, which are any untoward medical occurrences during an intervention, should be assessed in all randomized trials [8, 97]. In cancer cachexia trials, some AEs might be caused by the anti‐cachexia treatment, but the treatment might also improve the tolerance of anti‐cancer therapies, thereby reducing the same or other AEs. In many cases, it is difficult to ascertain the true cause or causes of the occurrence or severity of an AE, and monitoring usually did not include the suspected causes. To address these important questions, the only option in this review was to compare the rates and severity of AE in the intervention and control arms.

AEs include a wide range of symptoms, laboratory abnormalities and organ function disruptions, making comprehensive statistical analysis challenging. AEs are listed and graded in standardized documents like the CTCAE [98]. However, documentation and reporting of AEs in clinical drug intervention trials generally varies greatly among trials, from detailed descriptions in the methods to no mention at all [99]. Reports may range from extensive documentation of all possible disturbances to limited reports of prespecified or spontaneously occurring events. Statistical analysis is often hindered by inconsistent data collection, multiple testing and inadequate power [99].

In our analysis, 42 trials reported data on AEs, making them the second most frequently used oncological endpoint. As observed for other clinical trials, the collection, analysis and reporting of AEs were inconsistent, often limited by multiple testing and mostly being observed as non‐primary outcomes [99]. Most trials reported only a few selected AEs, sometimes limited to high‐severity grades, often referring to CTCAE Versions 3 and 4. Choosing AEs as an endpoint in cancer cachexia trials, which include anti‐cancer therapies, is valid, as reporting AEs is mandatory in randomized trials. Reducing AEs can improve QoL, tolerance of anti‐cancer therapy and possibly clinical outcomes. For meaningful statistical analysis, it is important to focus on pre‐selected AEs and adjust for multiple testing.

### 4.1 Significant Findings in Cancer Cachexia Trials

Upon reviewing the eligible trials, some reported statistically significant oncology outcomes. However, these findings are difficult to interpret with confidence due to several limitations. These limitations include among others large within‐trial heterogeneities of study participants, inattention to confounding factors, lack of efficacy of the cachexia therapy, selecting outcomes as non‐primary endpoints, lack of sample size calculation for primary outcomes and multiple testing. As a result, confidence in the validity of the reported statistical significance as well as the lack of it is low.

As OS is a key endpoint in cachexia as well as in oncology trials, trying to predict sample sizes in future CC studies targeting OS may benefit from recent oncology trial designs, for example, performed in patients with NSCLC. In studies comparing OS, HR between 0.7 and 0.8 are frequently targeted in trials performed in patients with NSCLC, either undergoing neoadjuvant combined chemo‐/immuno‐oncology therapy (HR 0.70) [100], receiving first‐line chemo‐/immuno‐oncology therapy in the metastatic setting (HR 0.76) [101], receiving palliative first‐line tyrosine kinase inhibitor treatment (HR 0.72) [102] or receiving second‐line palliative chemotherapy for progressive disease (HR 0.75) [103]. These HR are similar to those targeted in the here reviewed CC trials using OS as a primary endpoint by Rowland et al. [22] (HR 0.67), Baldwin et al. (HR 0.76) [39] and Bourdel‐Marchsson et al. (HR 0.74) [44].

Assuming a Type I error of 0.05 and a Type II error of 0.20 and aiming to detect differences in OS corresponding to HR of 0.70, 0.75 or 0.80 will require observing some 250, 380 or 630 deaths [104]. The sample size necessary to observe these events will depend on the mean survival of the control group, the follow‐up time and the censoring rate. Choosing a follow‐up of twice the mean survival time and assuming a drop‐out rate of 30% will require a total sample size of some 450, 680 or 1100 patients [104]. Shorter follow‐up and higher drop‐out rates will demand higher sample sizes, whereas smaller HR will require less patients. Stratification may prevent Type I error and improve power for small trials of less than 400 patients and may have an important effect for active control equivalence trials, but not for superiority trials [105].

Although the attempt to examine oncology outcomes is laudable, many critical considerations for determining outcomes in cancer research are often ignored in studies on cancer cachexia. For example, sufficient follow‐up for OS is frequently lacking, and the effects of subsequent treatments on survival outcomes are not adequately accounted for. Non‐cancer deaths, which can skew survival data, are also commonly overlooked. Additionally, there is a potential for bias in the assessment of disease‐free or event‐free survival, particularly in open‐label studies. Radiological assessments of objective and complete response rates often lack consistency, underscoring the importance of central reviews and ensuring that reviewers are blinded to study treatments. Moreover, there are varying definitions of surrogate endpoints, such as disease‐free survival and PFS, which can complicate interpretation. Symptom endpoints, or patient‐reported outcomes, present further challenges due to a lack of blinding, the risk of assessment bias—especially in open‐label studies—unvalidated instruments and inconsistent definitions. Symptom data are also prone to missing information and insufficiently frequent assessments. To address this, data collection for multiple symptom endpoints should be planned prospectively, with attention to hypothesis testing and necessary statistical adjustments specified in the study protocol. Unfortunately, these issues are almost universally overlooked in cachexia clinical trials. Two notable trials may serve as prototypes for addressing some of these challenges in cancer cachexia research. Temel et al. [48] studied anamorelin in a single tumour type—NSCLC—whereas Sandhya et al. [75] focused on a limited number of cancers, specifically in patients starting their first cycle of chemotherapy. By adopting these approaches, which control for potential confounding factors, the assessment of oncological outcomes may become more reliable and meaningful.

Future cachexia trials with oncology endpoints should prioritize focusing on a single tumour type, ideally at the same stage and undergoing the same cancer treatment. These studies should also be appropriately powered to ensure reliable results. Additionally, detailed and objective measures, such as body composition analysis, should be used to accurately assess the various aspects of cachexia. This approach will help minimize confounding factors and provide more meaningful insights into the impact of cachexia treatments on cancer outcomes.

The impact of anti‐cachexia treatments on cancer therapies is of considerable interest, as improving cachexia could theoretically enhance tolerance to cancer treatments or, by modifying tumour response, influence treatment outcomes and OS. However, examining this relationship robustly without the influence of confounding factors is challenging. Even when focusing on a single tumour type, the effects of different chemotherapy regimens can vary. For example, Klassen et al. studied various chemotherapy regimens for pancreatic cancer and found that their effects on muscle and fat differed significantly [106]. Additionally, factors such as tumour response and baseline BMI also affected changes in muscle and fat. This complexity suggests that rigorously assessing oncological outcomes in cachexia clinical trials remains challenging.

### 4.2 Strengths and Limitations

The strengths of this review include its rigorous methodology, such as the adoption of a prospective design (PROSPERO) and a thorough literature search covering the last three decades of cachexia trials. A robust strategy was used for appraisal and data extraction, involving multiple independent reviewers. The review utilized a validated quality appraisal tool (modified Downs and Black scale) and presented data to highlight the variety of studies using different oncological endpoints. The multinational and multi‐professional collaboration of experts ensured diverse inputs when evaluating the multidimensional condition of cancer cachexia.

However, the review has key limitations, including significant heterogeneity in patient populations, tumour types, definitions of cachexia and the various anti‐cancer and anti‐cachexia interventions. The different endpoints included in this review add to this heterogeneity. Although all analysed trials focused on assessing the outcomes of anti‐cachexia interventions, they did not compare different endpoints. This heterogeneity prevented a detailed assessment of the relationship between different endpoints, making it impossible to identify an ‘ideal’ oncological endpoint from the data. Nevertheless, the accumulated evidence from exploring oncological endpoints in depth can help guide the design of future cachexia trials.

## 5 Conclusions

Nutritional and metabolic endpoints, such as weight and muscle mass, are crucial in cancer cachexia trials. However, cachexia causes reduced survival and inferior deliverance of anti‐cancer treatment, and an effective cachexia treatment could consequently improve several standard oncological outcomes. Hence AE, completion of treatment and OS are clinically relevant endpoints, perhaps especially for patients undergoing anti‐cancer treatments.

Trials with oncology endpoints should prioritize focusing on a single tumour type, ideally at the same stage and undergoing the same cancer treatment. These studies should also be appropriately powered to ensure reliable results. Additionally, detailed and objective measures, such as body composition analysis, should be used to accurately assess the various aspects of cachexia. This approach will help minimize confounding factors and provide more meaningful insights into the impact of cachexia treatments on cancer outcomes.

## Conflicts of Interest

Richard J. E. Skipworth has received personal fees for consultancy from Artelo, Actimed, Faraday and Helsinn. Barry J. A. Laird has received personal fees for consultancy from Artelo, Actimed, Faraday, Kyona Kirin and Toray. Jann Arends has received personal fees for consultancy from Danone.

## Literature Search Strategy

1. Documentation on the literature search for ‘What is the optimal endpoint to evaluate effect of interventions aiming to treat cancer cachexia?’

**Table:**

The following databases were searched		
Database	Number of retrieved references for trials	Number of retrieved references for cohort/longitudinal studies
Medline (Ovid)	3812	1918
Embase (Ovid)	2033	2031
*Cochrane* Central Register of Controlled Trials	1923	
Number of references before de‐duplication	8166	3949
Number of references after de‐duplication	5998	3190

All searches were done 02 June 2021 by Gunn Kleven, Senior librarian at the Library of Medicine and Science, University of Oslo. These were then updated to cover the period from 2 June 2023until 17 October 2023 using the same search strategy as in the previous (until 1 June 2023).

Number of hours spent: 34

**Ovid MEDLINE(R) ALL** 1946 to 17 October 17

Date searched: 2 June and updated 17 October 2023 search strategy:

**Table:**

#	Searches	Results
1	exp Neoplasms/or (neoplasm* or cancer* or tumor* or tumour* or oncol* or malign* or carcinom* or adenocarcinom* or adenoma or metasta*).ti,ab,kf.	4 606 655
2	Cachexia/ or Emaciation/ or Malnutrition/ or Starvation/ or Wasting syndrome/ or Thinness/ or Sarcopenia/ or Anorexia/or *Weight Loss/	63 432
3	and/1‐2	9650
4	((cachexia or cachexic or anorexia or anorectic or emaciat* or malnutrition or underweight or starvation* or thiness or leanness or sarcopenia or wasting syndrome* or wasting disease* or weightloss* or ((appetite* or weight) adj2 (loss or loosing or losing))) adj4 (neoplasm* or cancer* or tumor* or tumour* or oncol* or malign* or carcinom* or adenocarcinom* or adenoma or metasta*)).ti,ab,kf.	7231
5	((cachexia or cachexic or anorexia or anorectic or emaciat* or malnutrition or underweight or starvation* or thiness or leanness or sarcopenia or wasting syndrome* or wasting disease* or weightloss* or ((appetite* or weight) adj2 (loss or loosing or losing))) and (neoplasm* or cancer* or tumor* or tumour* or oncol* or malign* or carcinom* or adenocarcinom* or adenoma or metasta*)).ti.	4253
6	or/3‐5	13 924
7	randomized controlled trial.pt.	536 354
8	controlled clinical trial.pt.	94 265
9	randomized.ab.	525 221
10	placebo.ab.	219 320
11	drug therapy.fs.	2 343 029
12	randomly.ab.	360 557
13	trial.ab.	557 904
14	groups.ab.	2 213 680
15	or/7‐14	5 047 938
16	exp animals/not humans.sh.	4 855 037
17	15 not 16	4 388 865
18	6 and 17	4078
19	limit 18 to yr = “1990‐Current”	3812
20	cohort studies/ or follow‐up studies/ or longitudinal studies/ or “national longitudinal study of adolescent health”/ or prospective studies/or retrospective studies/	2 168 707
21	(cohort* or longitudinal or prospective* or retrospective*).tw.	2 098 508
22	or/20‐21	3 012 965
23	and/6,22	3215
24	limit 23 to yr = “1990‐Current”	3139
25	24 not 19	1918
26	19 or 24	5730

**Embase Classic + Embase** 1946 to October 17, 2023

Date searched: 2 June and updated 17 October 2023 search strategy:

**Table:**

#	Searches	Results
1	exp neoplasm/or (neoplasm* or cancer* or tumor* or tumour* or oncol* or malign* or carcinom* or adenocarcinom* or adenoma or metasta*).ti,ab,kw.	6 400 926
2	cachexia/ or emaciation/ or *malnutrition/ or starvation/ or wasting syndrome/ or *anorexia/ or sarcopenia/or *weight loss/	93 608
3	and/1‐2	20 654
4	((cachexia or cachexic or anorexia or anorectic or emaciat* or malnutrition or underweight or starvation* or thiness or leanness or sarcopenia or wasting syndrome* or wasting disease* or weightloss* or ((appetite* or weight) adj2 (loss or loosing or losing))) adj3 (neoplasm* or cancer* or tumor* or tumour* or oncol* or malign* or carcinom* or adenocarcinom* or adenoma or metasta*)).ti,ab,kw.	9798
5	((cachexia or cachexic or anorexia or anorectic or emaciat* or malnutrition or underweight or starvation* or thiness or leanness or sarcopenia or wasting syndrome* or wasting disease* or weightloss* or ((appetite* or weight) adj2 (loss or loosing or losing))) and (neoplasm* or cancer* or tumor* or tumour* or oncol* or malign* or carcinom* or adenocarcinom* or adenoma or metasta*)).ti.	6437
6	or/3‐5	24 964
7	Randomized controlled trial/	666 248
8	Controlled clinical trial/	463 928
9	random$.ti,ab.	1 691 555
10	randomization/	91 413
11	intermethod comparison/	272 763
12	placebo.ti,ab.	330 754
13	(compare or compared or comparison).ti.	571 937
14	((evaluated or evaluate or evaluating or assessed or assess) and (compare or compared or comparing or comparison)).ab.	2 334 019
15	(open adj label).ti,ab.	88 467
16	((double or single or doubly or singly) adj (blind or blinded or blindly)).ti,ab.	251 401
17	double blind procedure/	187 924
18	parallel group$1.ti,ab.	27 750
19	(crossover or cross over).ti,ab.	112 863
20	((assign$ or match or matched or allocation) adj5 (alternate or group$1 or intervention$1 or patient$1 or subject$1 or participant$1)).ti,ab.	359 989
21	(assigned or allocated).ti,ab.	424 479
22	(controlled adj7 (study or design or trial)).ti,ab.	385 967
23	(volunteer or volunteers).ti,ab.	264 717
24	human experiment/	550 005
25	trial.ti.	340 818
26	or/7‐25	5 516 795
27	(random$ adj sampl$ adj7 (cross section$ or questionnaire$1 or survey$ or database$1)).ti,ab. not (comparative study/ or controlled study/or randomi?ed controlled.ti,ab. or randomly assigned.ti,ab.)	8736
28	Cross‐sectional study/not (randomized controlled trial/ or controlled clinical study/ or controlled study/or randomi?ed controlled.ti,ab. or control group$1.ti,ab.)	273 554
29	(((case adj control$) and random$) not randomi?ed controlled).ti,ab.	18 641
30	(Systematic review not (trial or study)).ti.	179 350
31	(nonrandom$ not random$).ti,ab.	17 184
32	Random field$.ti,ab.	2525
33	(random cluster adj3 sampl$).ti,ab.	1368
34	(review.ab. and review.pt.) not trial.ti.	902 488
35	we searched.ab. and (review.ti. or review.pt.)	37 332
36	update review.ab.	116
37	(databases adj4 searched).ab.	43 700
38	(rat or rats or mouse or mice or swine or porcine or murine or sheep or lambs or pigs or piglets or rabbit or rabbits or cat or cats or dog or dogs or cattle or bovine or monkey or monkeys or trout or marmoset$1).ti. and animal experiment/	1 113 420
39	Animal experiment/not (human experiment/or human/)	2 339 504
40	or/27‐39	3 735 626
41	26 not 40	4 907 360
42	and/6,41	3904
43	limit 42 to yr = “1990 ‐Current”	3674
44	limit 43 to conference abstracts	1641
45	43 not 44	2033
46	cohort analysis/ or follow up/ or longitudinal study/ or “national longitudinal study of adolescent health”/ or prospective study/or retrospective study/	3 499 550
47	((cohort adj (study or studies)) or cohort analy* or longitudinal).tw.	705 575
48	or/46‐47	3 722 608
49	and/6,48	4417
50	limit 49 to yr = “1990‐Current”	4387
51	limit 50 to conference abstracts	1639
52	50 not 51	2748
53	52 not 45	2031

***Cochrane* Central Register of Controlled Trials**

Date searched: 02 June and updated 17 October 2023

Search Strategy:

**Table:**

#1	[mh Neoplasms]	82 548
#2	((neoplasm* or cancer* or tumor* or tumour* or oncol* or malign* or carcinom* or adenocarcinom* or adenoma or metasta*)):ti,ab,kw (Word variations have been searched)	232 559
#3	#1 or #2	241 300
#4	[mh Cachexia] or [mh ^Emaciation] or [mh ^Malnutrition] or [mh Starvation] or [mh^“Wasting syndrome”] or [mh Thinness] or [mh Sarcopenia] or [mh Anorexia]	2665
#5	MeSH descriptor: [Weight Loss] this term only	6360
#6	#3 and (#4 or #5)	1017
#7	(((cachexia or cachexic or anorexia or anorectic or emaciat* or malnutrition or underweight or starvation* or thiness or leanness or sarcopenia or “wasting syndrome” or “wasting syndromes” or “wasting disease” or “wasting diseases” or weightloss* or ((appetite* or weight) near/2 (loss or loosing or losing))) near/3 (neoplasm* or cancer* or tumor* or tumour* or oncol* or malign* or carcinom* or adenocarcinom* or adenoma or metasta*))):ti,ab,kw	1475
#8	(((cachexia or cachexic or anorexia or anorectic or emaciat* or malnutrition or underweight or starvation* or thiness or leanness or sarcopenia or “wasting syndrome” or “wasting syndromes” or “wasting disease” or “wasting diseases” or weightloss* or ((appetite* or weight) near/2 (loss or loosing or losing))) and (neoplasm* or cancer* or tumour* or tumour* or oncol* or malign* or carcinom* or adenocarcinom* or adenoma or metasta*))):ti (Word variations have been searched)	758
#9	#6 or #7 or #8 with Publication Year from 1990 to 2021, in Trials	2345
